# Drug use and health behaviour among German men who have sex with men: Results of a qualitative, multi-centre study

**DOI:** 10.1186/s12954-016-0125-y

**Published:** 2016-12-09

**Authors:** Daniel Deimel, Heino Stöver, Susann Hößelbarth, Anna Dichtl, Niels Graf, Viola Gebhardt

**Affiliations:** 1 Catholic University of Applied Sciences, NRW/ German Institute for Drug and Prevention Research (DISuP), Aachen, Germany; 2 Department of Health and Social Work/Institute of Addiction Research (ISFF), Frankfurt University of Applied Sciences, Frankfurt/Main, Germany; 3 Faculty of Social Work and Health, Coburg University of Applied Sciences and Arts, Coburg, Germany

**Keywords:** MSM, Drug consumption, Chemsex, Club drugs, STI, HIV, Violence, Syndemic production

## Abstract

**Background:**

Men who have sex with men (MSM) are a risk group for new HIV infections. Drug use among men who have sex with men is often accompanied by risky sexual behaviours. Local AIDS help centres and gay advice centres are recording an increase in drug use among MSM clients in Germany. This study examines reasons for drug use and drug use contexts for MSM, including syndemic factors and experiences of social support.

**Methods:**

The study is based on a qualitative research approach. We conducted 14 structured, in-depth interviews with substance-using MSM in three German cities. An interview guidance document was used that was developed on the basis of the syndemic approach. Data analysis was based on structured analysis of content.

**Results:**

The MSM interviewed had extensive experience of drug use, in particular amyl nitrate, amphetamines, methamphetamine, ketamine, cocaine and cannabis. The drugs were used both at parties and in connection with sexual experiences (chemsex). Twelve men said that they had an existing HIV infection at the time of the interview. The men also reported experiences of violence and discrimination because of their sexual orientation. The social networks of the MSM and dating apps are highly relevant for experiencing chemsex. Certain places in the MSM community also have an impact on drug purchase, drug consumption and chemsex sessions.

**Conclusions:**

MSM are a group that is vulnerable to psychological problems, in particular problematic drug use. This group requires specific help from drug services and AIDS support services that are orientated towards the life situation and substance use contexts of the clients. Both support systems should work together more closely and network. Furthermore, specific prevention strategies, aimed at both mental and physical well-being, should be developed for substance-using MSM.

## Background

MSM are one of the main risk groups for new infections of HIV and other sexually transmitted infections (STI), both in Germany and internationally [1–3]. MSM still constitute the largest group of new HIV infections in Germany. Empirical results show a link between drug use and risky health behaviours, especially among MSM [4–8]. Based on comparative data collection from the last 20 years, it can be assumed that use of (illegal) drugs among gay and other men who have sex with men (MSM) is more widespread than in heterosexual comparison groups [9–12]. According to data from the German sample (*n* = 54,387) from the European MSM Internet Survey, the 12-month prevalence among MSM for amyl nitrate was 34%, 18% for cannabis, 6% for amphetamine, 3% for GHB/GBL, 3% for ketamine and 1% for methamphetamine [13].

MSM are valid as a vulnerable group for mental health problems and high rates of drug consumption [14, 15]. Main reasons for this are a negative internalised homonegativity (concealment of their own identity, fear of disapproval, etc.), a negative self-concept and also experience of discrimination and violence in the biography of the MSM [16–18].

The syndemic production approach [3, 19–24] and the minority stress model approach [25–27] give an explanation for the interaction of drug consumption, mental health problems, HIV and experience of violence in sexual minority populations. According to Singer [20, 226], syndemic describes “a concentration and deleterious interaction of two or more diseases or other health conditions in a population, especially as a consequence of social inequity and the unjust exercise of power”. As the first syndemic production, Singer [19] was able to prove the connection and interaction between drug abuse, experiences of violence and AIDS in the Puerto Rican community of Hartford. Social minorities are exposed to more intense strain and stressors, such as prejudice-based discrimination and experiences of violence, as well as internalised homonegativity. Drug use can be understood as the result of a dysfunctional coping mechanism and as the result of this increased minority stress [25].

There are two different situations that play a central role for drug consumption among MSM. Firstly, chemsex and, secondly, consumption of club drugs at parties. Bourne and colleagues define chemsex as follows: “sex between men that occurs under the influence of drugs taken immediately preceding and/or during the sexual session.” [28, 8]. In this case, substance use is particularly intended to provide a more intense sexual experience and improved sexual performance [29]. Intravenous drug use during sex (“slamming”), in particular, plays an important role in parts of the homosexual and bisexual community [30, 31]. As well as certain parties (e.g. circuit parties), use of dating apps and social networks in this context is, above all, significant for getting to know potential sexual partners [32, 33]. Local AIDS help centres [in Germany: AIDS-Hilfe] and gay advice centres are recording an increase in problematic chemsex among MSM in Germany [34]. Addiction support is not currently focused on the topic of sexuality and the everyday world of homosexual and bisexual men [35]. Furthermore, there is no empirical data on reasons for and contexts of use among substance-using MSM in Germany.

## Methods

### Ethics, consent and permissions

The study was subject to the data protection act of North-Rhine Westphalia (§ 28 DSG NRW). All participants provided written informed consent before interviews were conducted, and all identifiers were removed from the transcripts.

### Data collection

Because of the exploratory nature of the study, a multi-centre, qualitative research design was chosen. With the aid of structured, problem-centred interviews [36], substance-using MSM were surveyed about their experiences with drugs, the context of their drug use and their health behaviours. The subjects in the study were found through a local gay advice centre, an AIDS-Hilfe help centre and a specialist HIV practice, in three cities, Berlin, Cologne and Frankfurt am Main. There is an established homosexual and bisexual community in all three cities. The gay advice centre and the AIDS-Hilfe help centre provide psychosocial services, especially drug counselling, for MSM. They were founded by gay activists in the 1980s as self-help groups. Now they offer professional support from social workers and psychologists for this target group and are financed by local authorities. Homosexual and bisexual men who had experience of each kind of drug were spoken to. The men were recruited by social workers from help centres and by a doctor from an HIV practice. The men were having counselling in these institutions because of their drug use. The counsellors informed the MSM about the background and goal of the study. There were no other inclusion or exclusion criteria for the study. Systematic literature research [37] of relevant study results was conducted for developing the guidance document. The interview guidance document was based on the syndemic approach and the minority stress model [19, 20, 25]. We developed questions about the situation (health behaviour, drug consumption, etc.) and also questions about events in the biography (coming out, family life, etc.). The interview guidance document consists of 35 questions on six issues: sociodemographic data, drug consumption, sexual behaviour, biological family, experiences of discrimination and individual subjective meaning of the MSM community.

### Data analysis

The interviews were conducted at the advice centres’ facilities during the period from February to April 2015, recorded with a digital recording device and then transcribed using a transcription system [38, 39]. Because the interviewees were recruited from advice centres, the MSM interviewed considered their drug use to be problematic and had correspondingly sought help. It is therefore not possible to generalise the results to the population of MSM as a whole. The conversations recorded lasted between 20 and 70 min (50 min on average). The audio files were deleted after transcription for data protection reasons. Additional information was collected by the interviewer on a transcript sheet during the interviews and then annotated in another document after the discussion. The transcribed texts were systematically encoded using MAXQDA 11 analysis software and evaluated during a further stage as part of structured, qualitative content analysis. Mayring defines qualitative content analysis as a process “of systematically analysing texts, by processing the material in stages with theory-based category systems that have been developed on the basis of the material” [40, 114]. We used the following analysis process:

1. Development of a category system based on the syndemic approach and the minority stress model [19, 20, 25]
2. First analysis of the material
3. Development of additional categories
4. Second analysis of the overall material
5. Interpretation and evaluation of the results

During the process of analysis, we identified the meaning units that eventually became codes [41]. The category system includes seven main categories and 67 sub-categories. We found 1116 relevant codes in our analysis. The main categories were (1) socio-demographic data, (2) drug consumption, (3) experience of violence, (4) experience of discrimination, (5) sexuality, (6) help and support, and (7) MSM community. Encoding and evaluation of content analysis were each conducted by two researchers, in order to achieve a high level of consistency in the results. The researcher discussed the results of the encodings in regular team meetings.

## Results

### Sample description

The study sample consists of 14 men, six of whom lived in Cologne, five in Berlin and three in Frankfurt am Main. The age distribution clearly shows that the men interviewed tended to be older, with a high level of education (cf. Table 1). However, only five of the men obtained their income from a salary or self-employment. The majority (*n =* 10) was reliant on state benefits (sickness benefit, unemployment benefit or pension). Nine of the men interviewed lived alone in their own home. Three men lived with their steady partners, one man lived with his boyfriend in a shared dwelling place and one with another family member in a shared dwelling place.

**Table 1:** Participant demographic characteristics data

Age	26–35 years	36–45 years	46–60 years	
*n*	4	6	4	
Place of residence	Berlin	Cologne	Frankfurt/Main	
*n*	5	6	3	
School education	High school diploma/technical high school diploma	Qualification for admission to technical college	Secondary school leaving certificate	No school leaving certificate
*n*	8	2	3	1
Vocational training	Course completed	Vocational training	No vocational training	
*n*	7	5	2	

### Sexual identity, sexual activity and health behaviours

Nine of the 14 men describe their sexual identity at the time of the interview as homosexual or gay. Three men consider themselves to be homosexual, but mention bisexual tendencies.

That’s always so difficult to answer. I feel completely homosexual, but I have sex with women every now and then. I’m not interested in women, but I think they’re very pretty and beautiful. (Interview 5, line 283).

One man now describes himself as gay. However, he had been unable to develop a clear sexual identity until his 30th year. He found himself in relationships with women and fell in love with men. He describes the situation at that time as follows:

I basically saw myself as a sexless individual. I didn’t have a clue. Like that. (Interview 10, line 354–368).

A 50-year-old participant in the study was unable to clearly classify his sexual identity at the time of the interview. This was his reason for visiting a gay advice centre.

Na. I’m finding myself for the first time and trying to see what direction to take. (Interview 3, line 343–346).

Eleven men described their family’s reaction to their coming out as conflictual. Some parents reacted with a lack of understanding, some with clear disapproval or with reproach. One son was thrown out of the home. Another man was sent to have psychotherapy by his parents to have his homosexuality treated. Two men reported that their father became violent as a consequence of their coming out.

So I was really at loggerheads with my father, which then spilt over into physical violence and yes, then the youth office got me out. (Interview 7, line 204).

It became clear that mothers tended to be more likely to be able to accept their son’s homosexuality than fathers.

My mother reacted well to it, the rest of the family less so. They said “don’t touch my children anymore because you’ll turn them gay too” and grandad and grandma did not cope with it at all. (Interview 11, line 237).

Nine men were not in a steady relationship at the time of the interview. Four men were in a steady relationship, and one man reported that his relationship status was unclear.

Thirteen of the 14 men had been sexually active in the 12 months before the survey. The number of different sexual partners during this period varied between two and 200. The men can be divided into two groups: seven men reported a high frequency of sexual contact (more than 15 sexual partners/12 months, range 15–200 partners); six men reported a lower frequency (range 2–6 sexual partners/12 months).

According to their own information, 12 men were HIV positive at the time of the interview. Five men said that they had been infected with the Hepatitis C virus and two men had been infected with Hepatitis A or B in the past. Almost all men (*n =* 12) reported risky sexual situations in the past years, where condoms were not used. Two men described having incurred physical injuries during sexual activity.

Well, I managed to get my bowel ruptured with a toy (Interview 7, line 146–152).

Being infected with HIV, in particular, appears to lead to less use of condoms among some men. Some men reported conjoint, unprotected intercourse if their own viral load was below the detection limit or their sexual partner had the same HIV serostatus. One man reported consciously accepting HIV infection.

I just don’t like sex with condoms, I hate it. And, for that reason, I actually consciously decided to become HIV positive. Not because/so I just don’t like sex with rubbers. I think it’s disgusting and now, if you take tablets, antiviral treatment, you can’t infect anybody anymore, so if I take the tablets now and have sex with somebody, they can’t get infected, even if I bleed. It’s not possible. (Interview 1, line 281).

### Sex and drug use settings

The overwhelming majority of men reported that their alcohol and drug consumption was clearly linked with sexual activity. For one man, excessive alcohol consumption was important for coming into contact with other men.

Because, without alcohol, without being drunk, I won’t even go out in the first place, so I am always drunk when I go out. (Interview 3, line 365–372).

Eleven men reported that they had entered into risky sexual situations under the influence of drugs and that, if they took drugs, this tended to be what happened. In the view of the men, the reason for this was much poorer assessment of the risks of the situation, as well as a feeling of disinhibition.

I actually (…) took it for granted that sex on drugs was unprotected. (Interview 14, line 185–187).

Once, I think it was the first time I had really taken a lot of crystal meth. And then I was in a place, it was a “playroom”, in Schöneberg (Berlin), where/so you can hire it, it’s just for sex, with every possible toy and God only knows what. I was in there for three days and screwed every guy possible [Interviewer: three days non-stop?] Yes. And I tried everything I had wanted to try for a long time but never done, to protect myself. I am still HIV negative. I took all sorts of crazy risks and ran out and thought, oh shit, what was that all about? (Interview 2, line 179–183).

In the context of drug use, more excessive sexual behaviour combined with risky sexual practices was prominent among some of the men, in addition to unprotected sexual intercourse.

It becomes clear that acceptance of condoms is extremely low among some men. All in all, the men gave very different harm-minimising strategies for their sexual behaviour. They mentioned the following personal safer use and safer sex strategies:

- No sharing syringes or needles
- No direct blood contact during sex
- No sexual practices involving excrement
- Sexual practices only in safe places as well as closer contact with the sexual partner
- Sex only with known persons and in a personal milieu
- No anal intercourse in clubs
- No risky sexual practices (anal intercourse, fisting)
- Sex only with HIV-positive men (information from an HIV-positive man)
- Condom use
- Regular HIV and HCV tests
- Limit consumption of crystal meth during sex to only two sessions/year

The MSM interviewed did not say how often or regularly they used these strategies. The strategies mentioned only provide information about which subjective safer use and safer sex strategies the men have internalised.

**Fig. 1:**
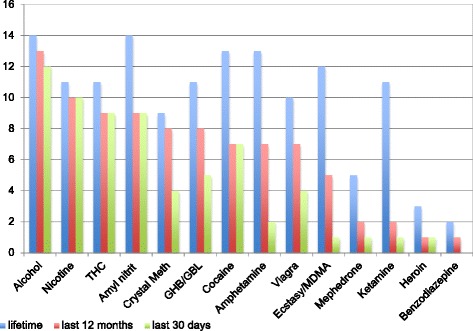
Prevalence of drug consumption

The interviewees have had numerous experiences with legal and illegal drugs (cf. Fig. 1). Most common are experiences with alcohol, nicotine, amyl nitrate, cocaine, amphetamine, ecstasy/MDMA, cannabis, GHB/GBL and ketamine.

Most men subjectively view alcohol as playing a secondary role in relation to other substances, even though it was consumed most frequently. All in all, 13 men had consumed alcohol in the last 12 months, 12 men in the last 30 days and two men consumed alcohol daily. This use was connected, in particular, with certain occasions or going out in the evening. Alcohol consumption was unproblematic for most of the men interviewed.

Nine of the interviewees said that they had recently experienced intravenous or intramuscular consumption of drugs. Methamphetamine (crystal meth) seems to be particularly important and was used intravenously by five of the users. Ketamine had been used intravenously by four of the interviewees, heroin by two and cocaine by one. Two of the interviewees reported rectal consumption of crystal meth and/or ketamine.

[Rectal administration:] people do it a lot with crystal meth, an awful, awful lot. Especially on the fisting scene. (Interview 05, line 179)

Crystal meth was the respondents’ preferred substance, mentioned most frequently by six of the interviewees now or in the past, but, for four men, it was equally as important as other drugs. They regard methamphetamine as the main drug, in conjunction with GHB (mentioned two times), cannabis and other amphetamines. The high importance is due to the intense rush of the substance itself that becomes even more significant when combined with intense sexual experiences.

Because the kick you get with this drug is the best kick there is. I’ve never tried heroin and I have never tried, you know, LSD or whatever, in my life, but this rush that you get when you inject it into the vein for the first time, it’s crazy. It’s just crazy, you just become a whore. All you do is have sex and all you think about is sex. What normal man can keep fucking for three days? Non-stop? Nobody. (Interview 1, line 195).

Three of the interviewees said that cocaine was the preferred substance. The connection with sexual activities is also particularly relevant in the case of cocaine. This also applies to the man for whom poppers is the most important substance.

Poppers, (laughs), there are thousands of bottles of it and I mainly just prefer this high. (…) During sex it makes you more relaxed and you have less will of your own. (Interview 8, line 75–79).

Four men said that cannabis was the main drug and one of them also said that alcohol was the most important substance to him. Cannabis use was for relaxation or for switching off from everyday life or was simply part of everyday life.

### Motivations for drug use

Different settings in which certain drugs are used by the men interviewed can clearly be seen. They select the substances according to their subjectively experienced suitability for the situation. Particularly stimulating substances such as amphetamine, cocaine or ecstasy are used at parties.

So I had / no, so […] the other drugs, ecstasy, speed, coke, you take them at home so that you can stay awake, so you can dance for hours and so that, in that setting […] it wasn’t a party without drugs, so drugs and parties and meeting friends and being cool and getting recognition from the others, that was the usual thing, all year round. (Interview 1, line 209–217).

So, it’s often the case that (…), so more something pure, just coke for dessert after the evening meal. Among my circle of friends it’s alcohol and coke. And more so at parties, because coke is not that practical. (…) And then more speed and pills [ecstasy] and GHB. (Interview 2, line 134–136).

Overall, drug consumption is very important in social situations and in contact with other users, their partner or other sexual partners. Some of the interviewees report use alone at home in relation to cannabis or alcohol. Drug use in the context of sexual contact is particularly highly relevant. In this context, it is mainly for breaking down inhibitions, intense experience of sexuality, as well as boosting sexual performance. Six men said that they could have sex for particularly long periods, from hours to days at a time, whilst on drugs.

Yes, sex without taking anything normally lasts 20 minutes maybe, half an hour, maybe three quarters of an hour. Sex on drugs, with him and with me, as it were, normally lasts for eight to ten hours. (Interview 13, line 130).

It is not possible to maintain a long-lasting erection after taking the substances consumed. Some men use Viagra specifically for this purpose. Three men report stronger sexual desire when on drugs. However, sex is also experienced more intensely on the whole, which is caused by a focus on the sexual experience, among other factors. Other issues cease to matter.

On drugs, sex just lasts much longer, it’s much more intense and I completely lose my sense of space and time. (Interview 14, line 116).

It’s just crazy, you just become a whore. All you do is have sex and all you think about is sex. (Interview 1, line 195).

Some users take drugs specifically to break down inhibitions, so they can abandon themselves to the sexual experience. This makes it easier for them to come into contact with other men or to take sexual opportunities or partake in practices that would be less easy or not even imaginable without taking drugs.

So you fairly quickly lose all of your inhibitions and you are really just blindly fixed on more, more of everything. More of the drug, more sex, more full-on sex, somehow you just want more of everything (Interview 2, line 223).

Giving complete expression to all aspects of your sexuality and many aspects of your sexuality just wouldn’t even be possible without these drugs. (Interview 1, line 225).

Drugs such as GHB, methamphetamine or ketamine, in particular, are sometimes used specifically to relinquish the ability to control oneself, as well as to lose inhibitions.

In many cases, it’s different. First, I no longer have control over my body and my partner. I just don’t care about what happens to me. I just let myself experience the feeling, that it is there and then, that the other one isn’t / that I am not as self-aware. Yes, that I have to be him, him or him, but I am just there and I like it. (Interview 5, line 245).

But it can also be worrying, as one interviewee reports in connection with use of ice spray (local anaesthetic).

In terms of thoughts, you are somewhere else entirely, but your head is completely free. You have absolutely no will of your own and perhaps no inhibitions.

[I: Passive or active?]

Passive. Actually just passive. You know longer really know what your body is doing. You totally lose all of your functions. And that actually wasn’t all that pleasant. (Interview 13, line 213–215).

For one of the men interviewed, alcohol consumption is important for entering the community in the first place. It helps him to break down social anxieties and insecurities, as well as feelings of guilt about his own sexual identity, and to tolerate his partner’s desire for other sexual partners.

I have now started to act a little, thank God, at 50 years old, a little more normally, for two years. I would like to just go out, I don’t want this hiding, hiding, hiding any more. Hardly anyone knows that I have sex with men. (…)

[I: That’s relatively late if you look at other people’s life stories.]

Yes, because I still have these anxieties. I can’t visit these groups if there are several people there together, because I have a panic attack. There are just so many things. Or even just with drugs or just with alcohol. Then I can do anything, no problem. (Interview 03, line 213–216).

Yes, that was easy/yes, then it isn’t as bad for me if I have messed around with some man or other, for example, or with my boyfriend, because he always wanted to fool around with other men. I didn’t know about that. I have always thought that relationships are relationships. (Interview 03, line 200).

Taking drugs changed sexual behaviour for many of the interviewees. They experience sex with many partners for many hours or for days. Their inhibitions are broken down and sometimes they allow practices that they would not allow when sober. Sexual desire and sexual practices are experienced more intensely as a result, including because of the complete focus. However, perceptions and expectations of satisfying sexuality are shifting. Some men describe this as a loss of any concept of “normal sex” and a strong association of sexuality with drug consumption.

As I get older, I don’t have to go out dancing every weekend anymore, but I would also like to regularly have the kind of sex that I imagine. And I need drugs for that. (Interview 2, line 586–588).

These intense, long-lasting sexual experiences cannot be achieved without drugs. There is therefore also a risk of relapse if abstinence from drugs has been achieved.

Incredibly intense (…) at some point I lost all concept of how long normal sex actually lasts. Because I had an extremely long relationship where we only took drugs for sex, at some point it became normal for me for sex to last for one and a half days and then five weeks ago I had this reason for a relapse. (Interview 14, line 116).

Other reasons for drug consumption were mentioned, along with references to sex. For example, drug use created a sense of community among users. It is practised by groups of friends and offers recognition, for example. Two interviewees experienced better self-confidence with drugs. The men also mention other problem areas. They reported using drugs to overcome anxieties, insecurities, and difficult life experiences or to deal with problems in general.

### Drug use, risky sex and protective strategies

The role of drugs within the community is described by all interviewees as being extremely important and, in some cases, inherent in the community since its beginnings. Most men strongly associate this role with going out (partying, dancing), sexuality and its expression within the community or both:

Yes, it’s more for partying, for sex. It’s mainly for sex, most people take it with sex. And yes, sex and parties. (Interview 6, line 291).

Let’s just say, if you want to have sex, sex without drugs is as likely as winning the lottery. (Interview 13, line 315).

According to the accounts of four men, drugs have again become more important in the community in recent years. They describe how the topic comes up increasingly often in their group of friends and in the community, whether it is actual consumption or discussions about it. Some interviewees talk about a kind of “normalisation” of dealing with drugs in sexual settings:

[…] but I know that you (…) just get onto the topic of drugs more quickly and what you do with them and you might even go more readily with a stranger to the toilet and do a line, because it’s somehow the right thing to do at the time. You meet, you have a nice chat, you’ve got a beer in your hand: “so, what are you on right now? You look wasted”. “Oh well, this and that. I’ll just come to the toilet with you”. (Interview 12, line 202 ff.).

Overall, the men tend to ascribe this increased importance to illegal drugs such as crystal meth, GHB or mephedrone, i.e. substances that are known for their effect in sexual contexts. However, the role is described differently in relation to local scenes. Berlin, in particular, is mentioned as the city where drugs are exceptionally valuable in the community:

Berlin, really massive community. In the beginning I went out there a lot, but I stopped, because I found it to be a bit like something that could swallow you up. It was a bit too intense for me, because, even in a normal café, where I had started chatting to someone, I had somehow got onto speed, ecstasy and other drugs after three, four or five sentences. (Interview 14, line 235 ff.).

The men also describe internet platforms as significant in this context. According to them, communication about drugs in connection with sex could be encountered more frequently there than offline.

[…] in almost every third chat where people want to meet, it’s all “are you taking something too?” and then you ask “what? - “yes, Tina or crystal, etc.” basically everything. (Interview 11, line 338).

Three men also express their concern about the increased importance of drugs in the community:

Because I come across the topic more and more frequently, I feel threatened by it, because I think that it’s becoming more common because more people are doing it. Somehow it’s coming closer to me, but I can’t put it into other words. I just have the feeling that it’s coming closer to me and I feel threatened by it. (Interview 14, line 215 ff.).

### The need for integrated support around drug use and HIV/STIs

The persons interviewed say that they have made use of different types of help and support. This help and support is provided by AIDS-Hilfe help centres, advice centres for gay men, advice on drugs with subsequent in-patient treatment, local doctors with treatment supported by medication, self-help (e.g. Narcotics Anonymous) and psychotherapy.

Something I have done again, at AIDS-Hilfe, is something called “emotional support”. And they came to me, but it wasn’t because of drugs at the time, because after the illness… (Interview 1, line 257).

This help and support is sometimes used separately, but sometimes combined:

So, at the moment, it’s out-patient treatment supported by medication, combined with support from the advice centre for gay men and medical support for treatment at the… (Interview 2, line 147).

Drug-related help focussed firstly on better control on self-regulation, substance-abuse programmes (KISS programme) and, secondly, on abstinence (through withdrawal, reduction and rehabilitation).

## Discussion

The men interviewed were able to look back on extensive experiences with drugs. Consistent with previous studies [28–31], our findings show that drug use is clearly linked to the men’s sexuality (function of enhanced sexual performance, feeling of a loss of inhibitions and more intense sexual experience).

Similar to further studies [32, 42], drugs also have a social function, in particular at parties, celebrations and during sexual activity. For some men, drugs have a higher function in dealing with problems (e.g. social anxieties, problems with self-esteem and treatment of pain). The individual substances are used in different settings depending on the anticipated effects. The men report many situations where they found themselves in risky sexual situations under the influence of drugs. In addition to loss of control through drug use, there was also a loss of control in sexual behaviour. This is seen, for example, in sexual practices in which the men would not have participated without the influence of drugs. Sustained consumption of different substances, as is practised in chemsex, is, in particular, to be regarded as harmful to health. Some men reported overdoses in connection with this.

The men exhibit many syndemic factors [19, 20] in their biographies, such as experiences of discrimination and violence, HIV infections and drug use, etc. (cf. Table 2). A causal link between drug use and these stress factors could not be reproduced because of the design of the study and the small sample size. Other studies could verify this connection [25–27]. We assume that the strong concentration of syndemic factors in the biographies of the MSM leads to a harmful combination. Nevertheless, when looked at under the approach of syndemic production, these MSM can be regarded as a vulnerable group [14, 15] for education about relevant psychological problems, in particular, addiction.

**Table 2:** Syndemic factors and health behaviour

Case	1	2	3	4	5	6	7	8	9	10	11	12	13	14
Sexual identity	Gay/bisexual	Gay	?	Gay	Gay/bisexual	Gay/bisexual	Gay	Gay	Gay	?	Gay	Gay	Gay	Gay
										Gay				
Conflicts in the family at coming out	Strong conflict	No conflict	Conflict	No conflict	Strong conflict	No conflict	Strong conflict	Conflict	Conflict	Strong conflict	Strong conflict	Conflict	Strong conflict	Strong conflict
Sexual partners last 12 months	20–25	20	15	20–50	100–200	50	2	3	5	0	N/A	6	3	4–5
Experience of violence	Situation of drug use and sex	No	Childhood	No	No	No	Father: coming out and sex	No	Situation of drug use and sex	No	Father: coming out	No	No	Father: childhood
Prostitution	No	No	Yes MSM	No	No	No	No	No	No	No	Yes MSM	No	No	No
HIV status	Positive	Negative	Positive	Positive	Positive	Positive	Positive	Positive	Positive	Positive	Positive	Negative	Positive	Positive
Hepatitis C infection	N/A	N/A	Positive	Negative	Positive	N/A	Positive	N/A	Positive	N/A	Negative	N/A	N/A	Positive
Hepatitis A/B infection	Hep. B positive	Infection not clear	Hep. A/Hep. B positive	N/A	N/A	N/A	N/A	N/A	N/A	N/A	N/A	N/A	N/A	N/A

The majority of the MSM interviewed did not identify themselves as addicts. Correspondingly, they did not normally attend advice centres specifically for addiction if they needed support because of their drug use. They received advice from local gay advice centres, AIDS-Hilfe help centres or local practices specialising in HIV, which are closer to the everyday world of MSM. From the point of view of gender-sensitive addiction work [43], cooperation with AIDS-Hilfe centres and addiction support institutions should be extended. Furthermore, specific advice and treatment options for drug-using MSM should be developed that are orientated towards the reasons and contexts for drug use and the living conditions of the men (e.g. home visits by programmes such as “emotional support” or programmes for reducing consumption and increasing control [44]). Lastly, support and help from self-help organisations is also relevant. In towns and cities where there is a large, established MSM community, specific prevention programmes should be put in place to cover topics such as “psychological health” and “violence prevention”, as well as the issue of drug use. The chemsex phenomenon is extremely multifarious and complex. Support and treatment of drug-using MSM therefore requires holistic consideration of psychological and physical well-being, as well as contexts of use. It is so far unclear which drug-using MSM are particularly vulnerable and how they can be successfully reached by prevention and support services. There is considerable need for research and development in relation to this.

The limitations of this study include the potentially limited representativeness of the interviewees. We can only make a statement about MSM who describe their own drug consumption as problematic. The MSM interviewed were therefore in drug counselling. We cannot describe the situation of MSM who estimate their own drug consumption as unproblematic. Furthermore, the small number of participants limits the results of this study. We need to validate our results in a bigger population in further research.

## Conclusions

Our study results show that drug using MSM exhibit additional health-related burden. Their motives for drug consumption are manifold. Besides an improved sexual performance, the coping of various problems play a key role. The consumption of drugs is associated with a risky sexual behaviour.

## Abbreviations

AIDS: Acquired immunodeficiency syndrome
GHB: Gamma-hydroxybutyrate
HCV: Hepatitis C virus
HIV: Human immunodeficiency virus
LSD: Lysergic acid diethylamide
MSM: Men who have sex with men
STI: Sexually transmitted infections
